# A case of pulmonary tuberculosis with multiple nodules mimicking lung metastases

**DOI:** 10.1259/bjrcr.20180124

**Published:** 2019-04-01

**Authors:** Kazuhiko Morikawa, Shigeki Misumi, Taiki Fukuda

**Affiliations:** 1 Department of Radiology, The Jikei University School of Medicine, Tokyo, Japan

## Abstract

Tuberculosis (TB) may present as multiple pulmonary nodules mimicking lung metastases. Many asymptomatic cases of TB are detected incidentally on chest radiography, and patients are often negative for acid-fast bacilli staining and culture in spite of having active TB. It is important to know the imaging findings characteristic of pulmonary TB and its variant forms. Multiple pulmonary nodules were detected in an 80-year-old female during a medical checkup. TB was not suspected until her imaging findings worsened; however, in retrospect, centrilobular micronodules were observed amongst multiple well-circumscribed nodules on the initial images and worsened during conservative management. Although bilateral multiple well-circumscribed pulmonary nodules are suggestive of metastases, when a nodule surrounded by centrilobular or satellite micronodules is found, even in the absence of characteristic findings such as cavitation or the tree-in-bud sign, pulmonary TB should be considered in the differential diagnosis to prevent delays in treatment.

## Case presentation

An 80-year-old female was referred with multiple pulmonary nodules discovered during a medical checkup. She had no symptoms of fever, cough, dyspnea, chest pain, or hemoptysis, but had noticed unintended weight loss of 5 kg in the previous 6 months. She had a medical history of Type 2 diabetes, hypertension, hypercholesterolemia, and ischemic stroke. She was a cigarette smoker (10 per day for 40 years). Physical examination revealed no abnormalities.

### Investigations

**Figure 1.**
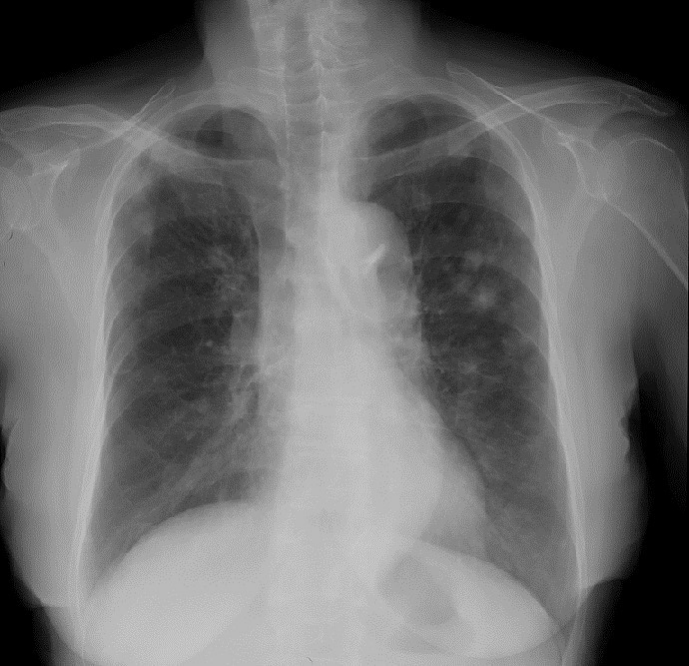
A Chest radiograph performed at the time of presentation showing multiple nodules bilaterally that are predominantly in the upper lung fields.

**Figure 2.**
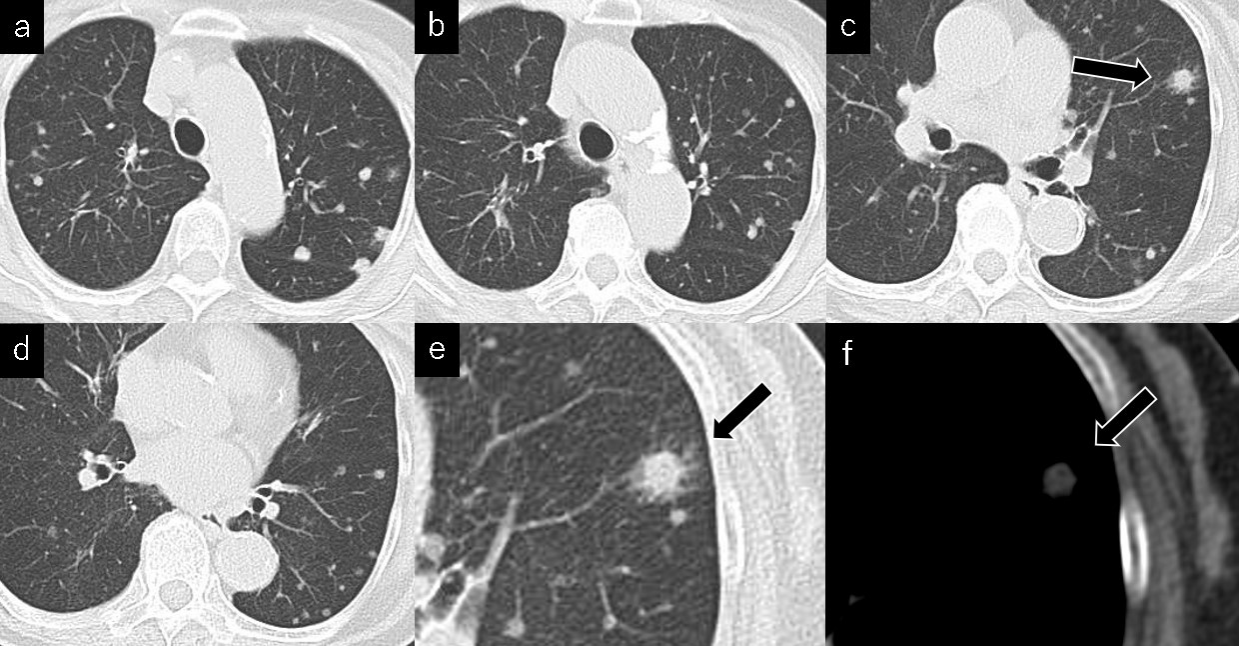
(a)-(f) CT images of the chest performed at the time of presentation showing multiple well-defined or slightly irregular-shaped nodules in both lungs. (e) A high-resolution CT scan obtained at the same level as (c) shows a large nodule surrounded by centrilobular or satellite micronodular opacities in the left upper lobe (arrow). (f) A CT scan on mediastinal window obtained at the same level as (c) shows a nodule with soft-tissue attenuation similar to that of muscle, and no calcification is identified (arrow).

Routine laboratory investigations revealed that her leukocyte, C-reactive protein, and hepatobiliary enzyme levels were within normal limits. Serum tumor markers, including squamous cell carcinoma antigen, carcinoembryonic antigen, cancer antigen (CA) 19-9, CA 72-4, α fetoprotein, and protein induced by vitamin K absence or antagonist II (PIVKA-II), were also within the normal ranges. Tests for hepatitis B surface antigen, hepatitis C antibody, and human immunodeficiency virus were negative. Chest radiography revealed multiple nodules approximately 2 cm in size bilaterally and predominantly in the upper lung fields (Figure 1). CT of the chest showed multiple well-defined or slightly irregular-shaped nodules with soft-tissue attenuation in both lungs (Figure 2). None of nodules contained calcification (Figure 2f). In consideration of multiple pulmonary nodules in an elderly female, the radiological diagnosis on initial CT was probable multiple lung metastases and tuberculosis (TB) was not considered. In retrospect, one of the lung nodules in the left upper lobe was noted to be surrounded by micronodular opacities (Figure 2c and e) and calcification of the hilar and mediastinal lymph nodes was detected (no image presented).

**Figure 3.**
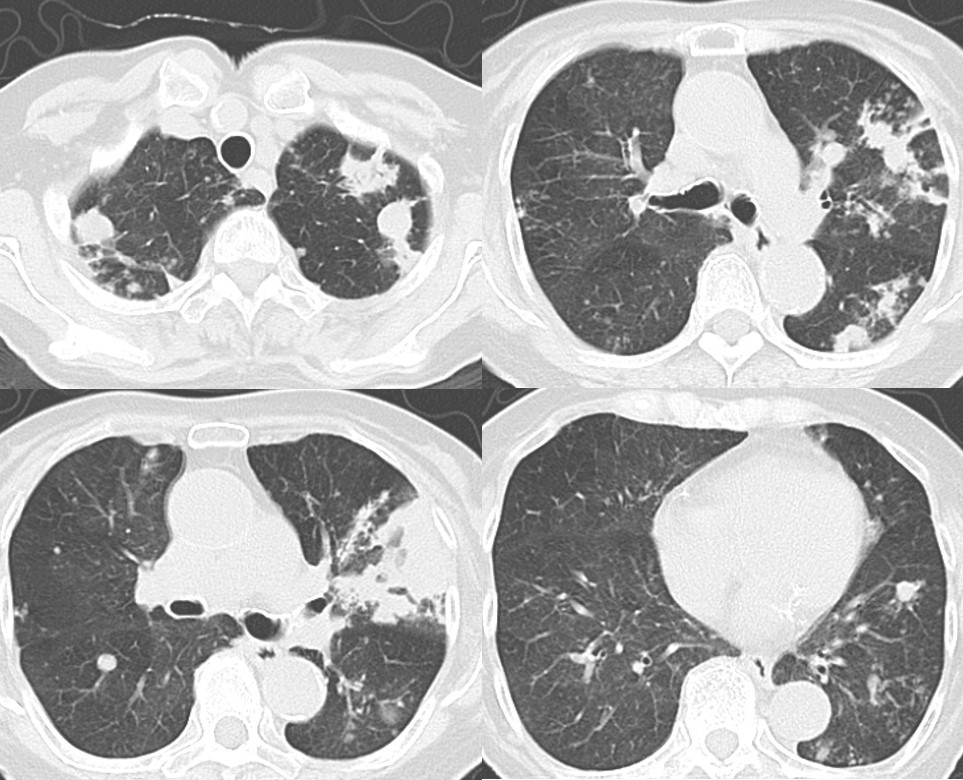
A CT scan of the chest performed 5 months after the first visit. 5 months after the first visit, dense consolidation and scattered micronodules became apparent.

Whole-body CT, gastrointestinal endoscopy, colonoscopy, and ultrasonography of the thyroid gland and breast were performed under the presumptive diagnosis of malignancy, but no obvious primary lesion was detected. Some of these investigations were performed on admission; at this time, an acid-fast bacilli (AFB) smear and *Mycobacterium tuberculosis* polymerase chain reaction (PCR) of a sputum specimen which were performed as screening tests were negative. Unfortunately, a T-SPOT interferon γ release assay was not performed. Given that the patient was an elderly female who did not wish to undergo further invasive examinations, such as bronchoscopy or percutaneous biopsy, the decision was taken to simply observe the pulmonary nodules. The size of the nodules gradually increased, and centrilobular and branching nodular opacities surrounding these enlarged nodules became apparent. 5 months after the first visit, consolidation and scattered micronodules appeared in the left upper lobe (Figure 3). Unlike acute bacterial pneumonia, the boundary between the consolidation and normal lung parenchyma was clear. Pulmonary TB was suspected in view of the findings on imaging of a chest shadow characteristic of caseous pneumonia and the tree-in-bud appearance.

### Differential diagnosis

In elderly people with multiple pulmonary nodules, a diagnosis of metastatic carcinoma is more likely than a diagnosis of TB. When multiple nodules accompanied by centrilobular or satellite micronodules are found, the differential diagnosis includes infectious disease (including TB and nontuberculous mycobacteria), sarcoidosis, and mucosa-associated lymphoid tissue lymphoma. The distribution of nodules in recurrent TB and sarcoidosis is predominantly in the upper lung zones. Although these diseases must be excluded, a radiologic finding of such nodules is more characteristic of TB and sarcoidosis than lung metastases. From a clinical point of view, patients who are immunocompromised, those with chronic renal failure requiring dialysis, and those with diabetes are at increased risk of developing active TB.

### Treatment

An AFB smear test and *M. tuberculosis* PCR of sputum specimens were found to be positive. A subsequent sputum culture also became positive for TB and a T-SPOT interferon γ release assay was positive. The patient was started on an anti TB chemotherapy regimen consisting of rifampicin, isoniazid, and ethambutol for 2 months and rifampicin and isoniazid for a further 7 months.

### Outcome and follow-up

**Figure 4.**
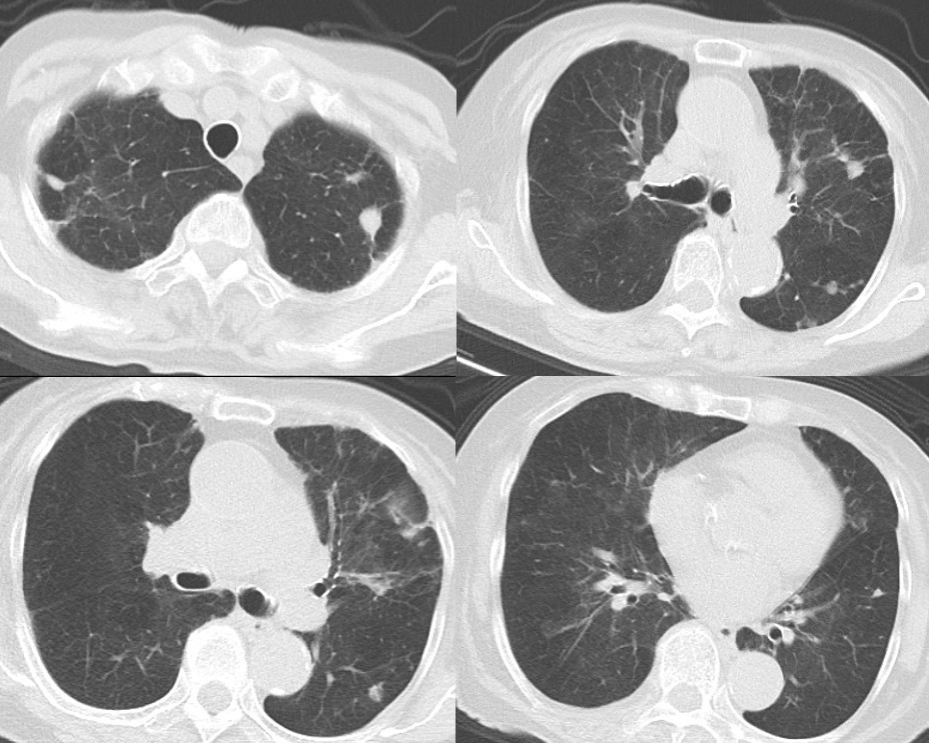
A CT scan of the chest performed 2 years after treatment. The pulmonary nodules and consolidation had resolved. Slight pulmonary scarring remained.

The thoracic shadow was observed to improve and finally an AFB smear test and sputum culture became negative for TB. At approximately 2 years after treatment, the pulmonary nodules had resolved and slight pulmonary scarring remained (Figure 4).

## Discussion

There are many asymptomatic cases of TB that are detected incidentally on chest radiography, and patients are often negative for AFB staining, culture, and PCR in spite of having active TB. In such cases, the radiologic findings are not typical of TB and may mimic a number of other diseases.^1,2^ A tree-in-bud appearance and cavitation are known to be typical findings on CT; however, TB may also present as a solitary nodule (tuberculoma) that resembles pulmonary carcinoma.^1–3^ Moreover, there have been sporadic reports in the English literature of pulmonary TB occasionally presenting as multiple well-defined nodules that mimic multiple lung metastases.^4–8^

Miliary TB results from hematogenous dissemination of tubercle bacilli and presents with multiple sharply or poorly defined micronodules measuring 1–4 mm bilaterally,^4^ which is quite different from the radiographic presentation in our patient.

A well-circumscribed nodule or mass caused by TB is known as a tuberculoma and is an uncommon entity that cannot be distinguished from a neoplasm based on imaging studies alone. It appears typically as a solitary, round, calcified, and sharply marginated opacity measuring 0.5–4 cm in size. A tuberculoma is usually found as a single nodule in an upper lobe; however, multiple tuberculomas are not uncommon.^4^ Pathologically, the central region of the tuberculoma consists of caseous necrosis and a marginal zone of epithelioid granuloma, inflammatory cells, and collagen.^3^ Pulmonary tuberculomas can be manifestations of both primary and post-primary TB and have been reported to be present in 6–9% of adult-onset and post-primary TB cases.^9^ Our patient might have developed post-primary TB because she had calcified lymph nodes in the mediastinum and hilum that could have been the result of previous TB infection. Moreover, not all tuberculomas are confirmed to be active TB by AFB smear or PCR tests of sputum and gastric juice, as was the case in our patient. Therefore, pathologic examination, such as percutaneous needle aspiration/biopsy or open thoracotomy, are often required to confirm TB.^1,4–7^

Several authors have reported that the most common CT findings on reactivation of pulmonary TB are small centrilobular nodules, branching linear and nodular opacities (the tree-in-bud sign), patchy or lobular areas of consolidation, and cavitation.^10^ The small centrilobular nodules and tree-in-bud sign indicate endobronchial spread and reflect the presence of caseous necrosis and granulomatous inflammation in and around the terminal and respiratory bronchioles and alveolar ducts.^10^ These radiologic findings are considered a reliable marker of the activity of the disease, and satellite nodules around the tuberculoma may be present in as many as 80% of cases.^10^

In retrospect, we realized that there were tiny nodules surrounding some of the larger nodules on the initial CT that should have raised suspicion for mycobacterial infection, including TB. Although TB rarely presents radiographically as multiple enlarging nodules masquerading as metastases, careful observation and detection of nodules surrounded by centrilobular or satellite micronodules may lead to the correct diagnosis. Unfortunately, the importance of such findings in differentiating between TB and lung metastases were not emphasized in previous similar reports.^4–8^ We believe this is an educational case highlighting the importance of the finding of centrilobular or satellite nodules around large nodules in the diagnosis of TB.

## Learning points

1. Pulmonary TB may present as multiple well-circumscribed nodules without transbronchial spread or cavity formation that mimic multiple lung metastases.
2. If there is a finding of a well-circumscribed nodule surrounded by centrilobular or satellite micronodules that worsens during conservative management, or multiple nodules with an upper lobe predominance, pulmonary TB should be considered in the differential diagnosis in patients with bilateral multiple pulmonary nodules. These small findings should not be overlooked.
3. Not all tuberculomas are confirmed to be active TB by AFB smear or PCR tests of sputum and gastric juice (as in our patient); therefore, TB should not be excluded even if it is not confirmed in the initial investigations. The investigations needed for a diagnosis of TB should be repeated, if there is a finding of a well-circumscribed nodule surrounded by centrilobular or satellite micronodules.

## Informed consent

Written informed consent for the case to be published (including accompanying images, case history, and data) was obtained from the patient.
